# Physical Activity and the Occurrence of Postnatal Depression—A Systematic Review

**DOI:** 10.3390/medicina55090560

**Published:** 2019-09-02

**Authors:** Daria Kołomańska-Bogucka, Agnieszka Irena Mazur-Bialy

**Affiliations:** Department of Ergonomics and Exercise Physiology, Faculty of Health Science, Jagiellonian University Medical College, Grzegorzecka 20, 31-531 Krakow, Poland

**Keywords:** postpartum depression, puerperium, pregnancy, physical activity

## Abstract

*Background and Objectives:* During pregnancy and the postnatal period many changes occur in a woman’s body, both in mental and physical spheres. The birth of a child and a new role—of a mother—can sometimes be associated with numerous negative emotions, uncertainty, fear, anxiety, disgust, depression, or sadness. In the puerperium period, the development of baby blues or postpartum depression may occur. Postpartum depression develops within one month of childbirth and may last up to one year. Depressive disorders that may develop in a young mother affect both her and the newborn’s health. That is why it is so important to try to search for factors that could significantly reduce the likelihood of developing depression in this period. The study aims at assessing the relationship between physical activity during pregnancy and puerperium or in the postpartum and the development of postnatal depression. *Materials and Methods:* A review of the literature was carried out in the Medline-PubMed database. The search terms were “pregnancy” AND “physical activity AND postpartum depression”. The study included only English-language publications published in the period 2000–2018. *Results:* A total of 216 references were found. After establishing the inclusion and exclusion criteria based on the analysis of titles and abstracts, 173 articles were excluded from the review. A total of 43 publications were read in full. Finally, 16 articles were included in the review. It was shown that regular physical activity during pregnancy, pregnancy, and puerperium, or in the postnatal period itself as compared to inactivity, reduces the risk of developing depression in pregnant women and after the birth of a child. *Conclusions:* Physical activity can be an essential factor in the prevention of depressive disorders of women in the postnatal period.

## 1. Introduction

Pregnancy, childbirth, and the postnatal period are some of the most important periods in women’s lives [1], in which there are changes in both physical and mental well-being [2,3]. In the postpartum period, lasting 6–8 weeks from the birth of the child [4], processes restore the woman to her pre-pregnancy status (e.g., uterus involution, postpartum wound healing, and increased abdominal muscle tension) [5]. Risk factors predisposing women to postnatal depression include, for example previous depressive episodes, feeling of anxiety and despondency in pregnancy, low self-esteem, poor partner relations, low socioeconomic status, and loneliness [6]. Also, women at risk of perinatal complications, hospitalization during pregnancy, or termination of childbirth by cesarean section after an earlier start of nature are also at risk [7]. Stress associated with caring for a child [8] and the lack of body acceptance after childbirth can also cause depression in women [9].

The first month of the postnatal period is very important due to the possibility of depressive disorders [10]. Three main depressive disorders may occur in women in the postpartum period (baby blues, postnatal depression, and postpartum psychosis) [11]. Baby blues (so-called postnatal sadness) [12] may develop four days after the birth of the child and last up to 12 days [13]. It was estimated that it could occur in 15.3% to 84% of mothers [14]. Baby blues is a risk factor for postpartum depression [15], which is 25% [13]. Postpartum psychosis is rare (0.1–0.2% of women after childbirth) [16].

Postpartum depression is defined as a serious mental disorder that develops within one month after childbirth [17]. Postpartum depression occurs in 6.5% to 20% of women in the postpartum period [18,19,20,21,22]. In the second month of the postnatal period, the risk of developing depression is 5.7%, while in the sixth month after childbirth it is 5.6% [23]. Anxiety, a sense of hopelessness, sleep disturbances, attention and appetite disorders, lack of interest in the child, and the environment are the main symptoms of postpartum depression [24]. In addition, suicidal thoughts may occur in severe cases [25]. Risk factors for postnatal depression include: Previous episodes of depression, depression and anxiety during pregnancy, self-esteem disorders, baby blues after childbirth, problems with partner relations, low socio-economic status, and perinatal complications [26,27]. Prematurity, low birth weight, or weakened functioning of the newborn are also predisposing factors for the development of depressive disorders in the postpartum [26]. Postpartum depression lasts from three to nine months, sometimes up to one year after the birth of the child [28]. Furthermore, depressive disorders in the postnatal period may have a negative impact on the development of the mother–child relationship [29].

The development of postnatal depression is influenced by hormonal changes in women during labor and postpartum [30]. Between the first and second stage of childbirth, progesterone concentration drops significantly, while after the expulsion of the placenta estrogen level decreases. Estrogen affects the levels of serotonin and dopamine due to the decrease in its concentration and has a negative effect on psychological well-being [31]. In addition, insufficient levels of estrogen and progesterone affect the development of anxiety disorders. For mood disorders, the posture is also more susceptible to sudden and rapid drops of B-endorphins [32].

A condition for diagnosing postpartum depression is the development of the disease directly related to the birth of the child [33]. The methods of treatment of postpartum depression include: Psychotherapy [34], the use of antidepressants [35], and hormonal therapy [36]. Additionally, light therapy [37] and exercise are also therapeutic interventions [34].

Physical activity has a significant impact on both the physical and mental state of pregnant women [38]. The American College of Obstetricians and Gynecologists recommends a minimum of 150 min of moderate physical activity within a week for pregnant women and after childbirth [39]. Exercise during pregnancy improves the return to form and minimizes the risk of postpartum depression [40], as well as reduces the symptoms of depression [41]. Physical activity in the postpartum period improves blood circulation, strengthens the abdominal and spine muscles, stimulates lactation, accelerates the constriction of the uterus, prevents urogynecological dysfunction, as well as improves the mental and physical condition of mothers [42]. The improvement of women after childbirth varies depending on the type of birth. In postpartum patients, a cesarean section may start physical therapy 10–12 h after birth [43] and after physiological labor 6 h [44]. Physical activity should be safe and adapted to the current state of the obstetrician [45].

The systematic review aims to show the relationship between physical activity and postpartum depression. In particular, the objective was to draw attention to the type, intensity, and duration of exercise to prevent or minimize the risk of developing depressive disorders in the postpartum period.

## 2. Materials and Methods

A literature review was carried out in the Medline-PubMed database. The primary search terms were “physical activity AND postpartum depression”.

### Search Strategy

((“exercise”[MeSH Terms] OR “exercise”[All Fields] OR (“physical”[All Fields] AND “activity”[All Fields]) OR “physical activity”[All Fields]) AND (“depression, postpartum”[MeSH Terms] OR (“depression”[All Fields] AND “postpartum”[All Fields]) OR “postpartum depression”[All Fields] OR (“postpartum”[All Fields] AND “depression”[All Fields]))) AND ((“2000/01/01”[PDAT]: “2018/12/31”[PDAT]) AND English[lang]). The study included English-language publications with randomization carried out and published in the years 2000–2018. The review was made according to PRISMA (Preferred Reporting Items for Systematic Reviews and Meta-Analyses) guidelines.

For the analysis of titles, abstracts, and comprehensive texts, inclusion and exclusion criteria were established. The review included works that were carried out and published in the years 2000–2018 in English. The examinations should have been carried out on pregnant and puerperal women, or only during the postpartum, and should have examined the relationship between physical activity and postpartum depression. In addition, only randomized controlled trials were included in the review. Exclusion criteria included studies that were published in a language other than English and were not related to women in the postpartum period. Studies were also rejected that were carried out among pregnant and puerperal women, or only in the puerperium, but not describing the impact of physical activity on the occurrence of postnatal depression. In addition, this study did not include studies that examined the impact of exercise on the development of postnatal depression, but it was impossible to determine the impact only on the period of the postnatal period more clearly. Research other than RCT (Randomized Controlled Trial) has not been included in the review. Bachelor’s theses, master’s theses, doctoral theses, letters to the editor, conference reports, and research protocols were also rejected. Other exclusion criteria included a lack of clearly described results and access to the full version of articles, as well as the lack of standardized scales to assess postnatal depression. Two independent researchers have verified the correctness of the adopted inclusion and exclusion criteria.

## 3. Results

**Figure 1:**
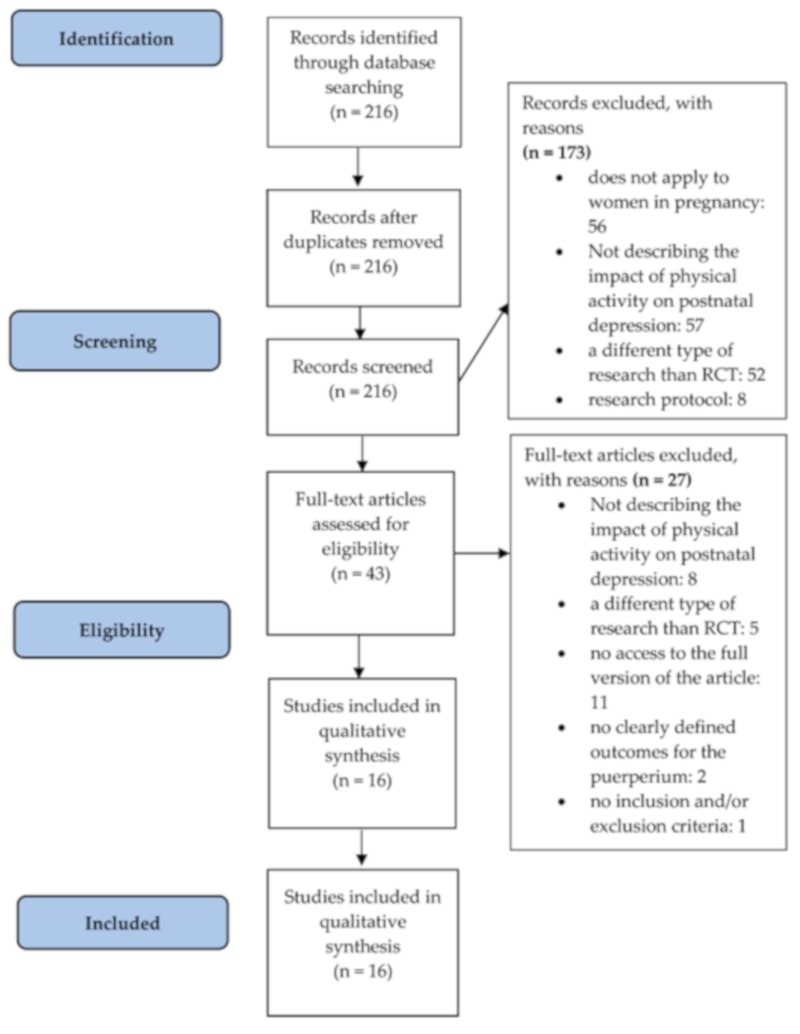
A diagram showing the review (2009 PRISMA-flow diagram stages of the literature).

In total, 216 references were found. A total of 173 articles were rejected based on the analysis of titles, abstracts, and the language of publication. A total of 43 articles were completely read. Finally, 16 studies were included in the review (Figure 1). The most important features of articles meeting the inclusion and exclusion criteria are presented in Table 1.

### 3.1. Exercises During Pregnancy, Or Pregnancy And Puerperium, And Postnatal Depression

The influence of physical activity on the level of depression during pregnancy and puerperium was examined by Vargas-Terrones et al. [46]. Women from the intervention group performed supervised training (60 min) three times a week, from 16–22 weeks of pregnancy to the end of the 3rd trimester. No intervention was implemented in women from the control group. The CES-D scale (The Center for Epidemiological Studies Depression Scale) was measured at the level of depression three times (beginning of the study, end of pregnancy, and six weeks of puerperium). Prior to the start of the project, there were no statistically significant differences between the groups in the level of depression. In the 38th week of pregnancy, the percentage of women with depression was significantly lower among physically active pregnant women compared to inactive women (respectively, 18.6% and 35.6%; *p* = 0.041). A similar result was obtained in the 6th week of postnatal period (14.5% experimental group (Exp. gr.), 29.8% control group (Con. gr.); *p* = 0.046) [46].

Similar studies were also carried out by Songøygard et al. [47]. Pregnant women between 20 and 36 weeks of pregnancy participated in supervised training (60 min, once a week for 12 weeks). In addition, in the last two weeks of pregnancy, they performed self-training at home for 45 min. The control group received standard care. The EPDS scale (The Edinburgh Postnatal Depression Scale) examined the level of depression three months after childbirth. Depressive disorders (EDPS ≥ 13) were found among four (1.2%) women from the intervention group, and eight (2.4%) from the control group (*p* = 0.25). Among women who were not physically active before the pregnancy occurred, two (2%) from the intervention group and nine (9.5%) from the control group obtained above 10 points in the EPDS score (*p* = 0.03) [47].

On the other hand, Daley et al. [48] examined the influence of supervised physical activity on the level of depression in pregnant women trying to quit smoking. Women from the intervention group received standard care and a training program. The control group did not perform exercises. During the training sessions, the women performed a 30 min supervised treadmill training (twice a week for six weeks and once a week for the next two weeks). Also, women were asked to increase their daily physical activity (especially walking) to 30 min by five times a week. The EPDS scale was used to assess depression. At the end of pregnancy, a significantly higher level of depression was obtained in the group of physically active women. Prior to the project, the mean level of depressive disorders in the control group was 7.7 (±5.0), and in the intervention group, it was 7.6 (±5.3). At the end of pregnancy, the control group experienced a decrease in the severity of depression (7.2 ± 5.0), and a group of active women increase by 8.0 ± 4.9. There were no statistically significant differences between the groups after six months of postnatal period (Con. gr.: 6.6 ± 4.7; Exp. gr.: 6.8 ± 4.8) [48].

The influence of exercises during pregnancy and postpartum depression on postnatal depression was also examined by Mohammadi et al. [49]. The training was carried out at home. The women who took part in the study were 26–33 weeks pregnant. They were divided into three groups: Control group; gr. I, exercise only during pregnancy; and gr. II, exercises during pregnancy up to the 2nd month of postnatal period. The level of depression was examined by the EPDS scale three times (before the start of the project, in the 1st and in the 2nd month). In 17% of women from the control group, 14% from a group I and 26% from group II were depressed (EPDS ≥ 13) in the first measurement. After the first month of the cycle, the following results were obtained: Control group: 12%; gr. I: 21%; and gr. II: 14%. After completion of the cycle (two months), depression was observed in 14%, 8%, and 8% of women in the control group, I, and II, respectively. No statistically significant differences between the groups were obtained at any point [49]. 

Aguilar-Cordero et al. [50] examined the influence of physical activity in the aquatic environment on the prevention of postnatal depression. Pregnant women from 20 to 37 weeks participated three times a week in water training according to the SWEP (Study Pregnant Water Exercises) method. The control group was not physically active. The EPDS scale examined the level of depression in the period between four and six weeks of the postnatal period. After the end of the cycle, a statistically significant lower level of depression was observed in the group of physically active women (Exp. gr. = 6.41 ± 3.68; Con. gr. = 10.17 ± 2.38) [50].

### 3.2. Physical Activity in the Postpartum Period and the Prevention of Postnatal Depression 

Le Cheminant et al. [51] examined the influence of resistance training in women in postpartum depression. Two different types of physical activity were compared. In the experimental group, women performed resistance training. During the first month, all training sessions were supervised, while in the 2–4 month, at least one session was supervised, and the remaining were recorded. The control group performed unattended stretching training. Before the beginning of the cycle, each woman received written instructions on how to perform stretching exercises and could participate in group training once a week. Symptoms of depression were measured by the CES-D scale before the project started, in the 2nd and 4th month. For women who performed resistance training, a significant decrease in the level of depression was recorded by comparing the initial analysis (9.5 ± 6.3) measurement made at four months showed (6.4 ± 4.1). A similar change was not observed in the stretching exercise group [51].

Lewis et al. [52] examined the impact of exercise and social support on preventing the development of postnatal depression among women at risk (*n* = 450). The study was attended by women who were at the time in their postnatal period (2nd day of the postpartum—11th week of postpartum) Also, before the birth of the child, they had depressive episodes. The examined women were divided into 3 groups: gr. I—physical activity, *n* = 150; gr. II—support, *n* = 150; and gr. III—standard care, *n* = 150. Lewis tested the physical training model at home for a minimum of 30 min, 5 times a week for 6 months. The subjects received instructional training on a DVD, which was thematically divided for the duration of the project. Group II also received a similar instruction, but concerning a healthy lifestyle in the postpartum period. Women of gr. III received standard postpartum care. For the duration of the research with women from the group, groups I and II were contacted by telephone by appointed consultants who supervised the project. The post-project evaluation showed a significantly lower level of depression in exercising women compared to the other two groups (*p* = 0.025). The level of fatigue was significantly lower in people who received standard care (*p* = 0.06) [52]. 

The influence of 12-week training on postpartum depression was also examined by Daley et al. [53]. A total of 38 women who had a child in the last 12 months qualified for the study. Two consultations on physical activity were prepared for the intervention group. The goal of the training was to increase the level of physical activity to 105 min a week. Women from the control group could not change their previous physical activity. There were no statistically significant differences in the level of depression between the groups. After 12 weeks, the average level of depression in the group of physically active women was 13.1 (±5.2), and in the control group it was 14.3 (±5.4). Depression was assessed using the EPDS scale [53].

Yang et al. [54] tested the influence of gymnastics on depressive disorders. A total of 140 women participated in the study in the 6th week of puerperium (Con. gr. = 70 and Exp. gr. = 70). Depression was examined using the EPDS scale. One section of physical activity lasted 15 min and was performed three times a week for three months. The women practiced at home using the instruction from a DVD. After a three-month cycle, a significant reduction in depression was achieved compared to the initial level in both groups (Con. gr. I analysis = 8.45 ± 4.68 and II analysis = 7.40 ± 4.46; Exp. gr. I analysis = 9.11 ± 5.54, and II analysis = 7.63 ± 4.81). A similar result was obtained by comparing the initial state with the results obtained three months after the end of the intervention in both groups (Con. gr. III analysis = 7.18 ± 4.54; Exp. gr. III analysis = 7.60 ± 4.71) [54]. 

Training at home using DVDs was also examined by Heh et al. [55]. The women were six weeks pregnant and had to exercise on the plates for 45 min, training twice a week. The control group did not receive any training. Depression was assessed on the EPDS scale twice: Before the start of the project and at the 5th month. In the 4th week of puerperium, no statistically significant changes were found between the groups in the intensity of depression (*p* = 0.83). After three months of training, the average level of depression in the experimental group decreased from 16.5 ± 2.6 to 10.2 ± 3.6, and in the control group from 16.3 ± 3.2 to 12.7 ± 3.9. Statistically significant differences in the level of depression were obtained between groups. [55]. 

Ko et al. [56] examined the influence of supervised aerobic training on the level of depression and fatigue in women in the puerperium. The training took place three times a week. Women who were assigned to the intervention group were obliged to participate in six training sessions for three weeks. The control group did not perform exercises. Depressive disorders were assessed using the CES-D. Before the beginning of the training cycle, the average level of depression in the group of active women was 14.13 (±5.12), while in the control group, it was 16.20 (±6.17). After the end of the project, depression was estimated at 12.42 (±5.37) and 14.53 (±6.94) points in the intervention and control groups, respectively. There were no statistically significant differences between the groups [56]. 

In contrast, Da Costa et al. [57] examined the impact of 12 weeks of home training on the level of postnatal depression. The study was attended by women who were at the time in their 4th to 38th week of the postnatal period. The level of depression was studied before and after the entire training cycle. The scale of EDPS and HAM-D (The Hamilton Rating Scale for Depression) were used to assess depressive disorders. The control group received standard postpartum care. During the 12 weeks, the intervention group met 4 times with a physiologist, with whom the intensity of physical exercise was increased. Women from the intervention group with an initial level of depression EPDS > 13 points achieved a significantly lower final result compared to the control group (average difference 4.06, *p* < 0.001). A similar result was obtained on the HAM-D scale (the average difference in points was 1.83, *p* = 0.02). After three months from the end of the project, the difference in the HAM-D scale was not statistically significant [57]. 

The influence of physical activity on depression was also examined by Forsyth et al. [58]. Before the start of the cycle, women from the intervention group consulted to increase their physical activity to 150 min a week. The control group could not change their current habits during the study. Both EPDS and SCID-PN (the Structured Clinical Interview for DSM-IV Axis I Disorders) did not significantly reduce the level of depression in the studied women. The average EPDS score before the start of the project was in the control group (15.9 ± 2.9), and after three months of supervision, it was 12.7 ± 4.2. But after the next three months from the end of the project, it was 12.7 ± 5.8. In the experimental group, the following results were obtained, respectively, at each subsequent measurement point: 17.6 ± 4.0, 11.8 ± 6.1, and 8.7 ± 6.9 [58].

Armstrong et al. [59] compared the impact of walking training and social support on minimizing depressive symptoms in women in childbirth. The EPDS scale examined the level of depression at 1, 6, and 12 weeks of the study. The marching sessions took place twice a week. Group II met once a week. During these meetings, women could discuss topics of interest to them. There was a statistically significant reduction in the level of depressive symptoms in physically active women (6 weeks: 10.2 points in the EPDS; and 12 weeks: 6.3 points). A similar result was not obtained for women from the gr. II (15 points on the EDPS scale in week 6, and 13.3 points in week 12). Before the beginning of the cycle in gr. I the average level of depression was 17.25, while in gr. II, it was 17.17 points [59]. 

Daley et al. [60] examined the influence of exercises on depressive disorders among 94 women in the postpartum period. Women in the 6th month of postnatal period qualified for the project. Depressive disorders have been studied using the Edinburgh Postpartum Depression Scale (EPDS) before the start of the project, and at the 6th and 12th month of intervention. During the weeks 1–12, the training lasted 30 min three times a week, while in the 13–24th week the frequency was increased to five times. The control group did not participate in physical activities. The mean level of EPDS depression examined in the 6th month was 12.51 (±5.46) and 14.67 (±4.86), respectively, in the group of active and physically inactive women. In the 12th month, however, an average of 12.02 (±5.29) points in the exercise group was obtained, and 12.55 (±5.17) in the control group [60]. 

Norman et al. [61] compared the impact of physical activity and education in the field of exercise on the level of postnatal depression. Women who were at the time in the 6–10th week of postnatal period were examined. The control group received educational materials related to a healthy lifestyle and care for a newborn for eight weeks. Women from the intervention group participated once a week for eight weeks in supervised training. In addition, they also received the same educational materials as women from the control group. After the end of the cycle, a statistically significant reduction in the level of depression in the intervention group was recorded compared to the control group (Exp. gr. = 5.47 ± 5.11; Con. gr. = 6.75 ± 5.51 points on the EPDS scale). Similar results were not recorded four weeks after the end of the project (Exp. gr. = 4.73 ± 5.26; Con. gr. = 6.54 ± 5.61 points on the EPDS scale) [61].

## 4. Discussion

Postnatal depression is the most common mental disorder of women recorded after the birth of the child [62]. In contrast, depressive disorders in this period may have long-lasting negative consequences both in the social, emotional, and cognitive dimension of the mother and child [63]. Unfortunately, despite the negative effects, postpartum depression is often undiagnosed and untreated. The main reasons for not treating depression are the fear of having a stigma of mental illness and the inability to breastfeed the baby due to the psychotic drugs used [64]. Research shows that a natural therapy that does not cause side effects is physical therapy [65]. Regular physical activities also eliminate the risk of developing depression during pregnancy and are a safe form of preventative treatment. Women who were also active before or during pregnancy had a lower risk of developing depressive disorders compared to physically inactive women (for a review see Kołomańska et al. [66]). According to the ACOG (American College of Obstetricians and Gynecologists) recommendation, physical activity should be resumed after childbirth as soon as possible and whenever it is safe [39]. Unfortunately, due to new responsibilities, many young mothers experience a decrease in the level of physical activity after childbirth [67].

The positive influence of regular physical activities on eliminating depressive disorders was explained using several mechanisms [68]. Exercises increase the concentration of neurotransmitters such as 5HT, dopamine, and noradrenaline [69]. In addition, physical activity increases the secretion of BDNF (a neurotic factor produced in the brain) [70], the concentration of which is low in people with depression [71]. The BDNF factor plays a very important role in the human body because it is responsible for neuroprotection, neurogenesis, and synaptic plasticity [72]. Exercises also increase cortisol levels [73]. Corticosteroids are responsible for the stimulation of the endocannabinoid system, which affects the action of such neurotrophins as BDNF [68]. Exercise also stimulates the production of GH (growth hormone) and IGF-1 (insulin-like growth hormone) [74]. GH and IGF-1 are responsible for the regulation of sleep, cognitive function, and mood. Moreover, it has been observed that in depression, there is an increase in the production of inflammatory cytokines, the concentration of which can also be reduced by regular physical activity [68].

Results from the evaluation of articles included in the review suggest that physical activity during pregnancy or puerperium may minimize the risk of development, as well as the symptoms of postnatal depression [46,47,48,49,50,51,52,53,54,55,56,57,58,59,60,61]. In a study conducted by Vargas-Terrones et al. [46], it was shown that regular physical activity in the second and third trimester of pregnancy significantly reduces the risk of developing depression both in pregnancy and in the postpartum. The influence of physical activity during pregnancy on the occurrence of depressive disorders in pregnancy and puerperium was also examined by Songøygard et al. [47]. There were no statistically significant differences in the occurrence of postnatal depression between the control and intervention groups. However, a lower rate of depression was observed among women who were not physically active before the project started and who were included in the intervention group compared to those in the control group. Lower levels of postnatal depression in physically active women in the aquatic environment during pregnancy were also observed in their studies by Aguilar-Cordero et al. [50]. The reduction of depression symptoms among women exercising during pregnancy, or pregnancy and puerperium was also noted by Mohammadi et al. [49]. Conversely, Daley et al. [48] obtained a statistically significantly higher level of depression among physically active pregnant women compared to inactive women. However, a similar result was not reported between groups at six months after childbirth. A meta-analysis performed by McCurdy et al. showed that light or moderate physical activity effectively reduces the symptoms of depressive disorders in women in postnatal period [75]. Similar results were also obtained in the analysis of Poyatos-León et al. [76]. In turn, Shakeel et al. conducted a cohort study among 643 pregnant women and showed that physical activity at the level of >150 MVPA (Moderate to Vigorous Physical Activity) min/week reduces the risk of developing postpartum depression compared to pregnant non-exercisers [77]. In the early puerperium period, physical activity with a mild degree of severity is recommended (walking, stretching exercises, and pelvic floor muscle exercises) [78]. LeCheminant et al. [51] noted that resistance training significantly reduces the symptoms of postpartum depression. However, this result was not obtained among women performing stretching exercises. On the other hand, Okyay et al. showed that physical activity not only minimizes the risk of developing postnatal depression, but also significantly improves the quality of life of women in childbirth [79]. The positive effect of 30 min training conducted five times a week on the level of depression was also noted by Lewis et al. [49]. A similar dependency was not demonstrated by Daley et al. [53] and Forsyth et al. [58] in which women were to be physically active for 105 min and 150 min, respectively, within a week. From studies on the impact of training using instruction from a DVD, it has been shown that exercises performed 2–3 times a week can significantly reduce the symptoms of postpartum depression [54,55]. A higher health awareness characterizes women who are physically active in the postpartum period compared to inactive women [80]. The impact of education on health and physical activity and education itself has been investigated by Norman et al. [61]. Immediately after the end of the project, there was a significant reduction in the level of depression among physically active women and those receiving educational materials compared to women who were in the control group [58]. The positive influence of long-term intervention (six months of training) on depressive disorders in women in puerperium was demonstrated by Daley et al. [60]. A similar result after 12 weeks of training performed at home was also obtained by Da Costa et al. [57]. Women in puerperium have increased the level of tension, anxiety, or fatigue [81]. Ko et al. [56] examined the influence of supervised physical training on the level of depression and fatigue in women in pregnancy. The intervention group showed a significant improvement in the level of fatigue compared to the control group. There is no similar dependence in case of depressive disorders [56]. Any type of postpartum training should be consulted with a specialist [43]. However, walking is the main physical activity that women start after giving birth [82]. It has been shown that walking training can significantly reduce the level of postpartum depression [59].

The presented studies show that physical activity is a significant element in the prevention and treatment of depressive disorders in the postpartum. Activities during both pregnancy and postpartum have a significant impact on the occurrence of postpartum depression. Only RCT-type tests were included in the review, which allows to compare the level of depression in at least two groups (control and experimental). Exercise groups were compared to physically inactive women, as well as those receiving social support, educational materials, or performing a different type of physical training. In all studies, the authors pointed out that physical activity is an essential element of a healthy lifestyle of women in childbirth.

## 5. Conclusions

In the recent years several systematic reviews have been published concerning the issue of physical activity and its impact on the development of postpartum depression [41,75,76,83,84,85]. The review contained in this paper is the most updated analysis of reference works, including literary resources up to 2018, whereas the previous systematic reviews were based on publications up to and including 2017 [41] or 2016 [75,76]. This review, taking into account RCT-type tests only, presents the holistic analysis of the influence of physical activity on the development of postpartum depression, without making a limitation in the types of activities, as in the paper of Pritchett et al. [83]—analyzing merely the aspect of aerobic training. As a result, it was possible to include a substantially larger number of works (16 articles) in this analysis, as compared with five [84] or six [85] analyzed in the previous review studies. 

Based on the review, it can be concluded that physical activity both during pregnancy, pregnancy and puerperium, or in the puerperium itself reduces the symptoms of postpartum depression. It also improves the quality of life and reduces the level of fatigue in young mothers. The supervised physical training, training in the water environment, as well as home-made exercises have a positive impact on the mental health of women in the puerperium. However, every activity should be started after prior consultation with a specialist and proper instruction. Mental and physical health are interdependent. Therefore, young mothers should be informed about the recommended level of physical activity. The correct development of a child depends on the woman’s well-being.

## Figures and Tables

**Table 1:** Study characteristics.

First Author (Year), Country	Title of Article	Main Objective	Sample Demographics	Research Tool	Main Results
Vargas-Terrones M. et al. (2018), Spain [46]	Physical exercise programme during pregnancy decreases perinatal depression risk: a randomized controlled trial	The impact of physical activity in pregnancy on the occurrence of depression during pregnancy and puerperium	Con. gr. = 54 Exp. gr. = 70	*CES-D*	There was a statistically significant difference between the groups in the depression level measurement both in the 38th week of pregnancy and in the 6th week of postnatal period. Exercises during pregnancy reduce the risk of postnatal depression.
Songøygard K.M. et al. (2012), Norway [47]	Does exercise during pregnancy prevent postnataldepression?	The impact of physical activity during pregnancy on reducing the risk of developing postnatal depression	Con. gr. = 340 Exp. gr. = 379	*EPDS*	There was no statistically significant difference in postnatal depression 3 months after childbirth between the two groups. Among women who did not exercise before becoming pregnant, but were included in the experimental group, there was a reduced risk of developing postpartum depression compared to the control group.
Daley A. et al. (2018), UK [48]	Physical activity for antenatal and postnatal depression in women attempting to quit smoking: a randomized controlled trial	The influence of LEAP (London Exercise and Pregnant Smokers) intervention on the pre-and post-partum depression	Con. gr. = 393 Exp. gr. = 391	*FTCD,* *EPDS,* *An accelerometer (Model GT1M or GT3X)*	During the last week of pregnancy, a statistically significant higher EPDS score was obtained in the physically active group compared to the inactive group. Statistically significant differences were not observed between the two groups in the measurements performed in the 6th month after childbirth.
Mohammadi F. et al. (2015), Iran [49]	The effect of a home-based exercise intervention on postnatal depression and fatigue: A randomized controlled trial	Evaluation of the effectiveness of stretching and breathing exercises performed at home to reduce postnatal depression.	Con. gr. = 42 Exp. gr. I = 43 Exp. gr. II = 42	*EPDS,* *FIF*	There were no statistically significant differences in the scale of EPDS and FIF between the groups after both 1 and 2 months of the intervention.
Aguilar-Cordero M.J. et al. (2019), Spain [50]	Moderate Physical Activity in an Aquatic Environment During Pregnancy (SWEP Study) moreover, Its Influence in Preventing Postpartum Depression	Effect of physical exercise during pregnancy on reducing the risk of depression in the postpartum period.	Con. gr. = 70 Exp. gr. = 70	*EPDS*, *RPE*, *A Quirumed OXYM2000 portable pulse oximeter*	It has been shown that women who performed exercises in water are less likely to develop postnatal depression compared to women who lead a sedentary lifestyle.
LeCheminant J.D. et al. (2014), USA [51]	Effect of resistance training on body composition, self-efficacy, depression, and activity in postpartum women	Effect of resistance training on the condition of women after childbirth.	Con. gr. = 30 Exp. gr. = 30	*PARQ*, *An Actigraph GT1M accelerometer*, *DXA*, *CES-D*	There were no statistically significant differences between the groups in the level of depression in all 3 measuring points (beginning of the study, 2 and 4 months). A statistically significant decrease in depressive symptoms was noted in the resistance training group. A similar result was not obtained in the control group.
Lewis B. A. et al. (2018), USA [52]	Rationale, design, and baseline data for the Healthy Mom II Trial: A a randomized trial examining the efficacy of exercise and wellness interventions for the prevention of postpartum depression	The effect of exercises and wellness interventions (sleep, stress, healthy eating) on preventing the development of postpartum depression in women at risk	Con. gr. = 150 Exp. gr. I (exercise intervention) = 150 Exp. gr. II (wellness/support) = 150	*SCID-I*, *EPDS*, *PSS*, *PAR,* *The ActiGraph motion monitor,* *PSQI,* *MFI,* *PSSQ,* *A 7-point Scale About Exercises,* *PHQ-9*	Physically active women showed a significantly lower level of depression compared to the other two groups.
Daley A. et al. (2008), UK [53]	Feasibility of an exercise intervention for women with postnatal depression: a pilot randomized controlled trial	Effect of exercises on the occurrence of postnatal depression.	Con. gr. = 18 Exp. gr. = 20	*The Godin Leisure-* *Time Exercise Questionnaire*, *EPDS*	There were no significant differences in the level of depression between the two groups.
Yang C.L. et al. (2018), Taiwan [54]	Effectiveness of aerobic gymnastic exercise on stress, fatigue, and sleep quality during postpartum: A pilot randomized controlled trial	The impact of aerobic training on stress, fatigue, sleep, and depression in women in the puerperium.	Con. gr. = 70 Exp. gr. = 70	*PSS*, *PFS*, *PSQS*, *EPDS*	After 3 months of the cycle, a significant reduction in depression was achieved compared to the initial level in both groups. A similar result was obtained by comparing the initial state with the results obtained 3 months after the end of the intervention.
Heh S.S. et al. (2008), Taiwan [55]	Effectiveness of an Exercise Support Program in Reducing the Severity of Postnatal Depression in Taiwanese Women	Evaluation of the effectiveness of physical activity to reduce the incidence of depressive disorders after childbirth.	Con. gr. = 40 Exp. gr. = 40	*EPDS*	In the 5th month of puerperium, a statistically significant reduction in the level of depression in both groups was noted. However, greater reduction was achieved in the group of physically active women.
Ko Y.L. et al. (2008), Taiwan [56]	Effects of Postpartum Exercise Program on Fatigue and Depression During “Doing-the-Month” Period	Effect of exercises on fatigue and depression in women after childbirth.	Con. gr. = 30 Exp. gr. = 31	*FSC*, *CES-D*	The level of fatigue decreased significantly between groups. Similar dependence was not obtained for the symptoms of depression. In the active group, a significant reduction in the level of depression during the study was demonstrated. A similar result was obtained in the control group.
Da Costa D. et al. (2009), Canada [57]	A randomized clinical trial of exercise to alleviate postpartum depressed mood	Evaluation of the impact of home-based exercises on lowering postnatal depression compared to standard care.	Con. gr. = 42 Exp. gr. = 46	*EPDS*, *HAM-D*	Physically active women showed significantly lower EPDS and HAM-D depression rates compared to women who underwent standard childbirth care.
Forsyth J. et al. (2017), UK [58]	Exercise as an adjunct treatment for postpartum depression for women living in an inner city—a pilot study	The impact of physical exercise on postpartum depression.	Con. gr. = 12 Exp. gr. = 12	*EPDS*, *SCID-PN*	There were no statistically significant differences in the level of depression between both groups in the 3 and 6 months of intervention
Armstrong K. et al. (2004), Australia [59]	The effectiveness of a pram-walking exercise program in reducing depressive symptomatology for postnatal women	The impact of exercise and social support on the reduction of postpartum depression symptoms	Con. gr. = 10 Exp. gr. = 9	*EPDS*, *SSI*, *RPE*	It was shown that, in physically active women, the level of their physical fitness improved, and the level of postnatal depression significantly decreased compared to the group of women in whom a social support program was introduced.
Daley A.J. et al. (2015), UK [60]	A pragmatic randomized controlled trial to evaluate the effectiveness of a facilitated exercise intervention as a treatment for postnatal depression: the PAMPeRS trial	Effect of exercises on the treatment of postnatal depression.	Con. gr. = 47 Exp. gr. = 47	*EPDS*, *CIS-R*, *SF-12SVS*, *IPAQ*, *The Actiheart Device*, *The Exercises Diary*	After 6 months of therapy, a significant reduction in the level of depression among physically active women compared to non-active ones was achieved.
Norman E. et al. (2010), Australia [61]	An Exercise and Education Program Improves Well-Being of New Mothers: A Randomized Controlled Trial	The impact of physical activity and health education on the mental well-being of women in the postpartum.	Con. gr. = 73 Exp. gr. = 62	*PABS*, *EPDS*, *Questions* *about the amount of physical activity*	A significant improvement in mental well-being and symptoms of depression in physically active women has been demonstrated compared to inactive women.

Legend: EPDS—the Edinburgh Postnatal Depression Scale; SSI—the Social Support Interview; SCID-I—the Structured Clinical Interview for DSM-IV Axis I Disorders; PSS—the Perceived Stress Scale; PAR—the 7-Day Physical Activity Recall; PSQI—the Pittsburgh Sleep Quality Index; MFI—the Multidimensional Fatigue Inventory; PSSQ—the Postpartum Social Support Questionnaire; PHQ-9—the Patient Health Questionnaire; CES-D—the Center for Epidemiological Studies-Depression Scale; FTCD—Fagerström Test for Cigarette Dependence Score; FIF—the Fatigue Identification Form; PARQ—the Physical Activity Readiness Questionnaire; DXA—X-ray absorptiometry; PFS—the Postpartum Fatigue Scale; RPE—the Borg Rating of Perceived Exertion Scale; FSC—a Fatigue Symptom Checklist; HAM-D—the 17-item Hamilton Rating Scale for Depression; PABS—the Positive Affect Balance Scale; CIS-R—the Clinical Interview Schedule-Revised; IPAQ—the International Physical Activity Questionnaire; SCID-PN—the Structured Clinical Interview for DSM-IV, Perinatal Version; SF-12—the 12-Item Short-Form Health Survey; SVS—the Subjective Vitality Scale.
